# Inclusion of librarians and information professionals in Canadian knowledge synthesis grant funding

**DOI:** 10.29173/jchla29701

**Published:** 2024-04-01

**Authors:** Brianna Henshaw, Rachael Bradshaw, Aubrey C. Geyer

**Affiliations:** 1 Reference and Instruction Librarian, Douglas College, Vancouver, BC, Canada; 2 Rehabilitation Sciences Reference Librarian, University of British Columbia, Vancouver, BC, Canada; 3 Liaison Librarian, University of British Columbia. Vancouver, BC, Canada

## Abstract

**Introduction:**

Librarians continually advocate for the expertise they can bring to knowledge synthesis research projects. Professional associations like the Canadian Health Libraries Association aim to promote librarians and information professionals as partners in health research. This push for representation must happen at a policy level to enact change. To that purpose, we explored the degree to which the inclusion of librarians and information professionals is represented at the funding level by healthcare research organizations in Canada.

**Methods:**

We used a list of health research funding agencies generated from Scopus searches and an independent search of Canadian health research institutions, governmental health authorities, professional associations, and research-oriented universities to identify research grants designed for knowledge synthesis research. We examined these grants to determine whether they require or specifically mention librarians in their eligibility criteria.

**Results:**

Of the 14 knowledge synthesis grants we identified, only one required a health librarian as a member of the research team in the grant eligibility criteria. Four grants “strongly recommended” the inclusion of librarians on the research team, though this inclusion was not a contingency for funding.

**Discussion:**

Most knowledge synthesis grants in Canada do not require, recommend, or mention librarians as members of the research or authorship team. Evidence suggests that librarian involvement substantially improves the quality of knowledge synthesis research projects; it would therefore benefit both librarians and knowledge synthesis work to advocate for librarian involvement as a contingency for grant funding.

## Introduction

The medical and health fields have long relied on various forms of knowledge synthesis to gather and interpret existing information to provide high-quality evidence that can be used to inform medical decisions and policy [1]. Systematic reviews and other forms of knowledge synthesis have risen greatly in popularity in recent decades to become some of the most prominent and highly trusted forms of medical evidence [1]. Systematic reviews are considered the “gold standard in evidence synthesis,” and as such, “influence important decisions such as patient care and resource allocation” [2]. As the popularity of knowledge synthesis has grown, so has the demand for its publication. Publications in the medical field are expanding at a pace commensurate with that of other academic fields, demanding more and higher quality synthesis work to mediate the burden of the massive amounts of published literature available for practicing professionals and scholars [3]. The increase in publishing volume is consistent across many disciplines including and beyond the sciences, and potentially reflects the increasing pressure that academics face from their organizations to consistently publish their works to meet career benchmarks or to increase their publishing metrics rather than merely an increase in the quantity of research being created [3].

This massive increase in the volume of published scholarship demands a greater number of rigorous, high-quality knowledge synthesis publications. However, various studies have raised concerns about the quality of published systematic reviews. In a study of systematic intervention reviews on the topic of children with cerebral palsy, Kolanski et al. found that 88% (*n=73*) of the 83 systematic reviews that they examined scored critically low on their AMSTAR-2 overall confidence ratings due to serious flaws such as exclusion of relevant studies, failure to establish review methods prior to conducting their reviews and/or to justify deviations from their protocol, and inadequate search strategies [4]. Errors in search strategies appear to be quite common, with one study showing that 92.7% (*n=127*) of the 137 systematic reviews examined contained some type of search strategy error, while 78.1% (*n=107*) of reviews contained errors that affected recall [6]. However, it appears that this issue can be aided or even solved by the involvement of librarians or other information professionals in knowledge synthesis work [6]. Henceforth in this article, we will refer to all information professionals as “librarians” for the sake of brevity.

Many studies have found that a librarian co-author contributes to higher quality search strategies in systematic reviews [7-10]. Koffel found a strong association between the use of recommended search methods in a systematic review and librarian involvement, as well as a positive association between librarian involvement and the quality of the search in a published systematic review [7]. Librarian involvement in systematic reviews is also associated with the use of a wider array of information sources, such as subject-specific and regional databases, gray literature, and other web searches when compared to systematic reviews without librarians [8]. Systematic reviews with librarian involvement not only have frequently improved search strategies, but also improved reporting processes. These projects tend to score higher on reporting elements of the review, such as the “study inclusion process, the date that the search was updated, [and] the full search strategy” [8]. Rethfelsen et al. similarly found that systematic reviews with librarians as co-authors use more methods that reduce bias than both reviews that simply mention librarians as contributors and reviews that do not include a librarian [9]. Thus, librarians are more likely to search for “non-English publications,” search in “regional database and subject databases… [and] gray literature” and to hand search for “conference proceedings and relevant journals,” all of which contribute to a more robust systematic review [9].

The importance of librarian involvement is recognized in the literature and by leaders in the field of evidence synthesis. The Cochrane Collaboration, the Centre for Reviews and Dissemination, and the Joanna Briggs Institute all recommend including an information professional either through consultation or as a member of the review team [11-13]. One of the most influential funding agencies supporting knowledge synthesis in Canada, the Canadian Institute for Health Research (CIHR), strongly recommends the inclusion of “an information scientist or librarian” in their *Knowledge Synthesis – Tips for Success* [14].

In addition to the work that they perform in service of science, librarians have long been motivated to develop key competencies in knowledge synthesis research [15]. Knowledge synthesis is a key point in raising the profile of librarians’ academic services, creating partnerships with researchers, and adding value to their institutions [16,17]. The increasing prevalence of knowledge synthesis research in and beyond the health and medical fields has offered opportunities for librarians to step into additional research roles in academic and special libraries [3,4]. Professional associations such as the Canadian Health Library Association (CHLA) urge librarians to seize these opportunities to be involved through their mission and vision statements, which state that they wish librarians to be “valued partners in the improvement of health, health care, research, and education” [18]. Increasing research partnership opportunities for librarians is, of course, a secondary goal to the refinement of quality in reviews. However, it is nevertheless important for librarians to ensure their contributions leverage their skills and expertise to represent and promote themselves as key contributors to the research process on behalf of themselves and their profession.

Many librarians spend considerable amounts of time advocating for their own value and expertise within the teams, departments, and institutions in which they find themselves embedded. This advocacy work is especially important because of the fact that many researchers seem to be unaware of the significant value that librarians bring to knowledge synthesis work. Several studies have found that while librarian involvement in published systematic reviews is growing, acknowledgement of the role librarians play in such reviews is still low [7-8, 10]. Amongst the 495 articles used in their review, Meert et al. identified only 11% that mentioned a librarian in the review process [8]. This number increased to 22% following a survey sent by the authors on librarian involvement [8]. While these numbers are low, they also indicate that librarians are more involved in knowledge synthesis work than is acknowledged in the published literature. Schellinger et al. similarly found that only 2.5% (*n=23*) of the 913 systematic reviews they identified on dental medicine included a librarian as a coauthor, while 9% (*n=82*) acknowledged librarian involvement somewhere in the review [10]. Koffel contacted authors about their systematic review publications and found that only 51% (*n=739*) of reviews reported librarian involvement in any capacity [7]. Among those that involved a librarian, only 64% (*n=485*) acknowledged said librarian, either through authorship (26%, *n=195*), in the text (8%, *n=63*), or in the acknowledgements section (33%, *n=251*) [7]. However, in “25% of [cases] where the librarian created or executed the search, their contributions were not recognized in the publication” [7]. This seeming lack of understanding and subsequent lack of acknowledgement of librarians and their roles in knowledge synthesis is troubling It led us to wonder if and how librarians are recognized by the organizations and grants that fund knowledge synthesis projects. That curiosity became the impetus of this research article.

The *Strategy for Culture Change*, articulated by the Center for Open Science, states that the final step to enacting behavioural changes is to “make it required” at a policy level [19]. Librarian involvement in knowledge synthesis may begin at a personal level, but it must be taken up by larger organizations such as professional associations, research institutions, and funding agencies for librarians to become normalized and acknowledged participants in knowledge synthesis work. Funding often determines the scope of a research project, the number of researchers who can be on the research team, and other personnel variables. Librarian involvement in knowledge synthesis research – and indeed any research – is only sustainable as long as there exists the funding to support their work. It should not be “off the side of the desk” work. Organizations should understand that this work is being completed regardless of a librarian’s official job description or the existence of pay structures to support such work, whether it is because a librarian is passionate about contributing to health research, because they are trying to embed themselves into a research team, or because the research team itself acknowledges the value a librarian can bring to their research and seeks them out. By investigating whether grant funding incentivizes or requires librarian involvement in knowledge synthesis work, we aimed to determine to what degree librarians are recognized by funding agencies as “valued partners” in this aspect of health research [18]. For this project, we sought to identify to what degree librarians are represented at the policy level by examining the eligibility criteria for knowledge synthesis grant funding.

### Research question

Our project was guided by the following research question: to what degree are the contributions of librarians in published knowledge syntheses recognized and advocated for by research funding institutions in Canada through their policies, as evidenced by the publicly available eligibility criteria?

## Methods

We began by identifying Canadian funding agencies for health-related knowledge synthesis publications indexed in Scopus from the past five years. We chose to use Scopus as the funding agency filter allowed us to access funding metadata without reviewing individual papers, simplifying the data extraction process. This was followed by an independent internet search for additional organizations that fund or support health research conducted by all three authors based on the Scopus list, personal experience with research funding processes, and recommendations from our course instructors.

To construct the Scopus list, the search phrase “systematic review” was used as a stand-alone, and the results limited to 2017-current (including some early publication of 2023 documents, as this search was conducted in November 2022).^2^ We determined that “systematic review” was an appropriate search term due to the prevalence of published systematic reviews as compared to other forms of knowledge synthesis. In addition, the Scopus search only provided a base for our identification of organizations, which would be supplemented by independent searching. Instead of reviewing individual papers for their funding sources, Scopus filters were employed. First, we used the Scopus filter for country/territory, which refers to the publication location for the journal. This approach offered the best method for narrowing the list to Canadian publications and, consequently, Canadian funders. No disciplinary filters were applied, as systematic reviews are primarily limited to the health fields, and any non-health-related funding sources would be eliminated in review. This list of organizations was extracted to a spreadsheet for review, where we performed manual deduplication and identified the geographical location of the funding bodies.

Organizations identified during the independent search were primarily: Canadian headquarters of international companies from the Scopus list; provincial and territorial research institutions, health authorities, and work safety boards; and the U15 Group of Canadian Research Institutions. This independent search also produced a list from the Nova Scotia Health Authority that identifies health research funding from Canadian medical associations and research institutions [20].

We conducted an initial screening of the Scopus list and our independent search by categorizing each organization into its institution type and its geographical jurisdiction. We grouped the institutions that we reviewed into categories that we created based on what type of institution they are using the following titles: corporation, governmental organization, NGO, university, association, public research agency, research institute, and non-profit. We excluded organizations that were not based in Canada (including international corporations with a Canadian subsidiary) and those that did not address topics in biomedicine, healthcare, or health-related topics on their public website. We also removed duplicate entries and organizations that are now defunct.

Our secondary screening addressed whether the selected organizations offered funding for knowledge synthesis research. This funding consisted primarily of research grants. Grants that included “knowledge synthesis,” “systematic review,” “meta-analysis,” “scoping review,” and/or “rapid review” were included in this stage, which we will refer to using the umbrella term “knowledge synthesis.”. Although we identified a large number of grants that could be used to fund knowledge synthesis research, we decided to only include grants which directly addressed knowledge synthesis topics either in the grant title or in the introduction, scope, or eligibility criteria for the grant, and excluded any that did not use the above terms to describe themselves, or which were specifically for primary or clinical research. This allowed us to focus on grants that were specifically designed with knowledge synthesis research in mind. We also excluded grants that were more than five years old (2017) for which we could not find a more recent version of the grant.

Using this list of grants, we assessed whether the eligibility criteria of the grant mentioned a librarian. We investigated whether the grants we reviewed required librarian involvement as per the eligibility criteria of the grant, whether the funding institution mentions or recommends that a librarian be involved in part or all of a knowledge synthesis project, or whether librarians were not mentioned by either the grant itself or the funding institution. Each list of organizations and grants was divided into three equal sections. After coming to a consensus on definitions, each of the authors performed an individual assessment of one-third of the list. Any organizations or grants about which the authors were unsure were discussed as a group to form an agreed-upon consensus.

## Results

**Fig. 1:**
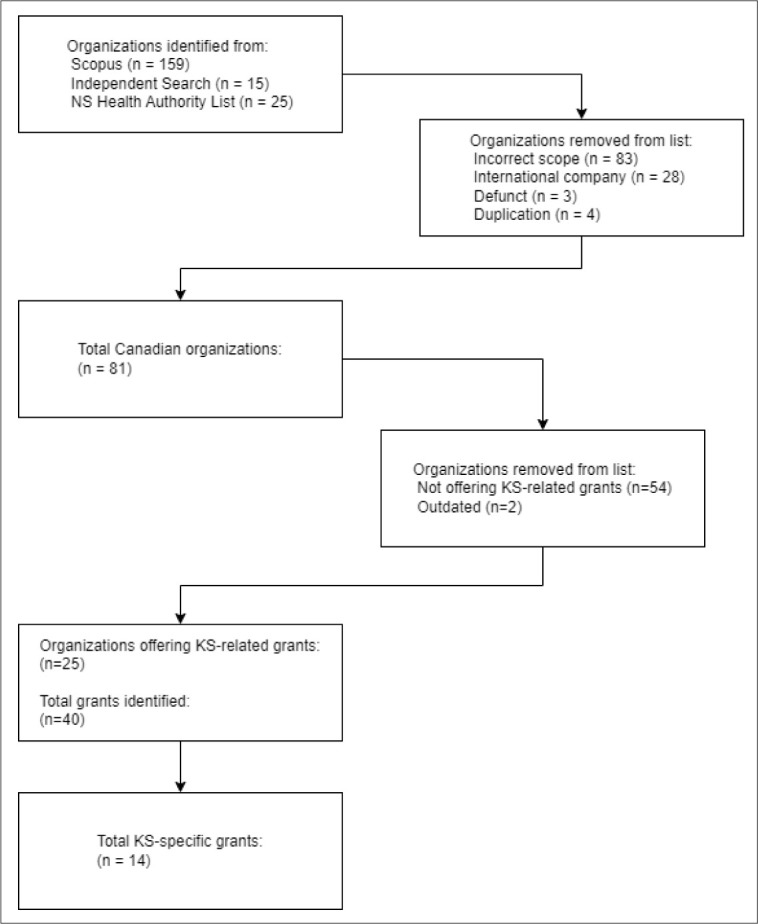
Flowchart of organizations and grants identified throughout the study

Our search for grants was conducted in November 2022. The resulting list of grants in Scopus contained approximately 22,000 papers, with records under the individual “Funding Agencies” filter ranging from 28 (several tied agencies) to 2,385 (CIHR). The 12,260 “undefined” records illustrated the limits of the metadata for this category. The final number of organizations pulled from Scopus was 159, which was extended through handsearching. A summary of the number of organizations included and excluded at each stage can be seen in Figure 1.

**Fig. 2:**
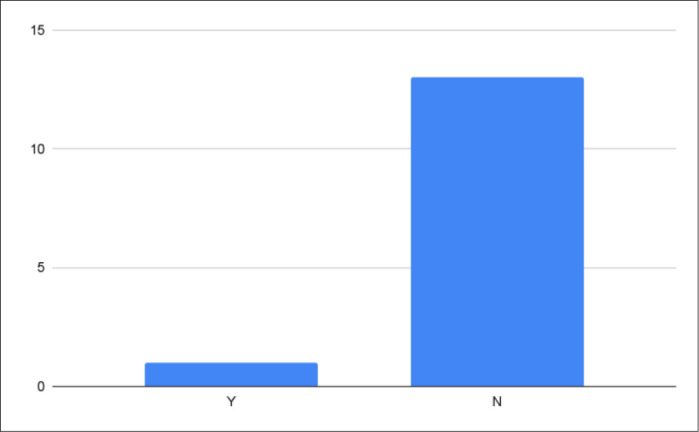
Mention of librarian or information professional in knowledge synthesis grants

We identified 14 knowledge synthesis grants from seven Canadian organizations. One grant mentioned the inclusion of a librarian or information professional in the eligibility criteria of the grant (see Figure 2). The “2022 Systematic and Scoping Review Grant Competition” from Alberta Health Services requires that “the review team must… include a medical librarian” [21].

In addition to reviewing the grants themselves, we also examined the general guidance for knowledge synthesis research – in other words, guidance related to knowledge synthesis creation in general, rather than tied to a specific grant – that our seven grant-funding organizations provide. Five of the seven organizations did not mention librarians. CIHR strongly recommends that knowledge synthesis researchers seek out the expertise of a trained information professional to support the development of a search strategy [14] and Alberta Health Services requires that a medical librarian be included on the research team [21]. For a full list of knowledge synthesis grants included in this study and to what degree librarians are mentioned by funding organizations, see the Appendix.

**Fig. 3:**
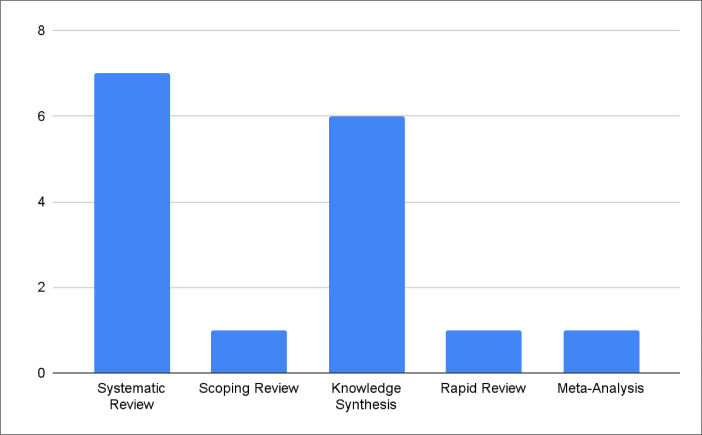
Number of grants per type of knowledge synthesis^3^

Our review of current knowledge synthesis grants in Canada provided additional insights. We found that the most common knowledge synthesis grant offered was for a systematic review (*n=7*), followed by general knowledge synthesis reviews (*n=6*), which are defined as grants that fund knowledge synthesis research without dictating which specific type of review is required to receive funding, then scoping reviews (*n=1*), rapid reviews (*n=1*), and meta-analyses (*n=1*) (see Figure 3).

**Fig. 4:**
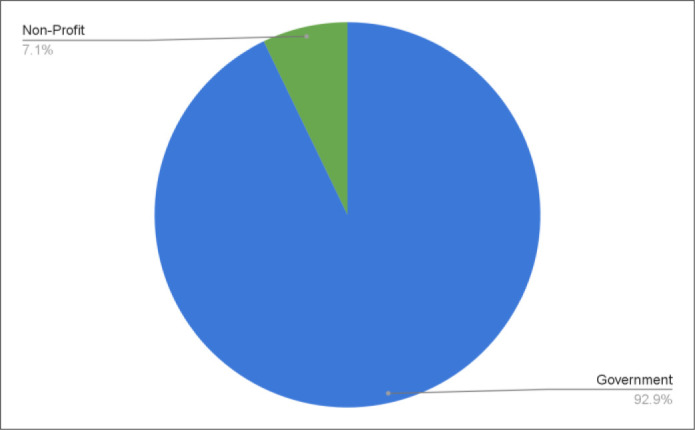
Grant funding by institution type^4^

**Fig. 5:**
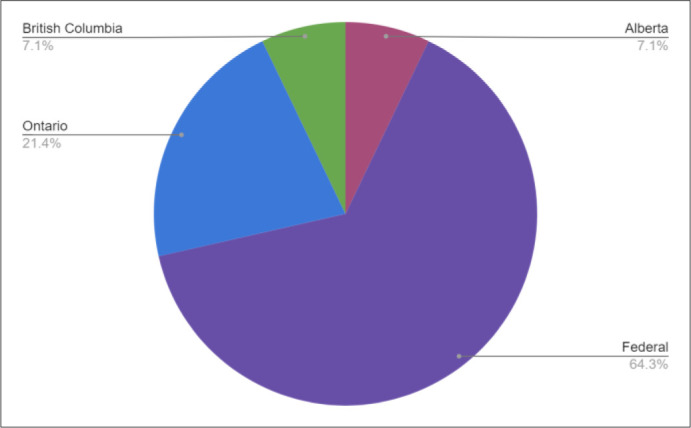
Grant funding by jurisdiction

**Fig. 6:**
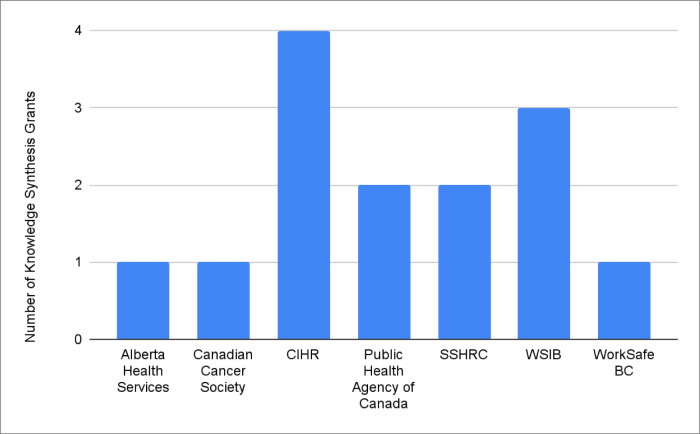
Grant funding institutions by number of knowledge synthesis grants^5^

Governmental organizations provided 93% (*n=13*) of knowledge synthesis grants, with 7% (*n=1*) offered by a non-profit (see Figure 4). Federal organizations were also the most common grant provider (*n=9*), followed by Ontario (*n=3*), Alberta (*n=1*), and British Columbia (*n=1*) (see Figure 5). CIHR provided the greatest number of grant opportunities (*n=4*), followed by the Ontario Workplace Insurance and Safety Board (*n=3*) (see Figure 6). See the Appendix for the institution type and jurisdiction of each funding organization.

## Discussion

The results of this funding survey reveal that only one of the 14 knowledge synthesis grants examined for this project required that a librarian be a member of the research team to meet the eligibility criteria. Four other grants, all from the CIHR, also “strongly recommend” that research teams include librarians. While some of the nine remaining grant applications mentioned other collaborators who could be involved in the research process, they did not specifically mention librarians (or any similar title) amongst their examples. Despite health librarians’ many and positive roles in knowledge synthesis research, there appears to be a lack of recognition at the funding level for the value that librarians add to knowledge synthesis in the majority of the grants that we reviewed.

Health librarians bring clear and demonstrated value to knowledge synthesis projects, as evidence demonstrates that having librarians involved at a higher level positively contributes to the quality of the review [7, 8]. In particular, embedded librarians may add even greater value to knowledge synthesis teams. Brander and Pawliuk state explicitly that the scoping review on which they worked “was enhanced in quality and efficiency through the integration of librarians onto the team,” going so far as to recommend that librarians be embedded into the research team from the outset of the project to assist with the scaling of the project and the creation of inclusion and exclusion criteria [31].

The lack of librarian acknowledgement by funding agencies seems to be at odds with the demonstrated recognition of their value both in published literature and by institutional leaders in the field of medical research. Librarians are explicitly mentioned by the CIHR and Alberta Health Services as recommended members of knowledge synthesis research teams in their general knowledge synthesis guidance [14]. Despite this acknowledgement of librarians’ value to knowledge synthesis research, none of the four grants that we identified from CIHR required librarian involvement as a stipulation for funding, nor even mentioned librarians as possible co-contributors to knowledge synthesis projects [22]. Organizations such as CIHR, the Social Sciences and Humanities Research Council (SSHRC), and other funding agencies often provide additional guidelines and definitions about potential acceptable collaborators who could be involved in a funded research project. Despite meeting the outlined requirements of these descriptions, librarians are not named as potential collaborators or other author types in the guidelines of any organization which we reviewed for this project [22-30]. Though granting agencies seem to recognize the contributions of librarians in theory, their roles do not appear to be valued in the execution of research through the grants that these agencies provide.

It is both out of our scope and difficult to say exactly why health librarians’ roles are under-recognized at at the funding level, though this is a promising area for future research. It is possible that to some degree librarians’ contributions to knowledge synthesis are undervalued due to a lack of awareness of the expertise that they bring to these projects, despite acknowledgement from bodies such as CIHR. In our experience, when librarians are involved in knowledge synthesis research, it is often because the review team is personally aware of the value that librarians bring to their project, rather than an external requirement. However, this value is not universally understood by health science researchers. Without an understanding of their value, researchers are unlikely to put aside the funds necessary to hire a librarian to be part of their team, potentially to the detriment of the quality of their knowledge synthesis project and discouraging further librarian involvement. It is helpful for the CIHR to recommend that librarians be involved in knowledge synthesis work in their guidelines, but without policy change at the funding level, the involvement of librarians in this work remains a suggestion that may or may not be heeded by researchers. Gaining researcher buy-in for the inclusion of librarians in their knowledge synthesis projects is certainly more complex than merely requiring them for grant funding. However, this inclusion has the potential to clear the path for librarian involvement among researchers who may not otherwise consider including a librarian as part of their team, making grant requirements a powerful advocacy tool for health librarians who wish to be involved in knowledge synthesis publication. It would greatly benefit both librarians and researchers who publish knowledge synthesis papers if the CIHR and its fellow research funding agencies were to push for librarian involvement in a more formal manner. Though the lack of requirement for librarian inclusion at the funding level is certainly a hurdle for librarian involvement in knowledge synthesis projects, it is not an insurmountable one. Health librarians must do what they can to highlight the value that they bring to knowledge synthesis, especially as these types of studies increase in popularity and demand in scientific and medical publishing. Through negotiating authorship with research teams, publishing articles about their involvement in knowledge synthesis, and publicizing their work through outlets like social media, health librarians can bring more attention to their work and make their involvement a priority for future knowledge synthesis funding.

## Limitations

We have identified several limitations in this paper. Primarily, it was written as the final project for a health information and services course, meaning that it has a limited scope, was written during a limited period of time, and did not have a budget. One such limitation was our use of Scopus for identifying funding agencies in our initial screening. While a broader selection of knowledge synthesis literature in the health sciences may be indexed in medical databases such as MEDLINE, CINAHL, or Embase, the funding metadata in Scopus was more accessible in the scope of a student project and the technical knowledge of the authors. A broader selection of initial knowledge synthesis research may generate additional funding agencies that we missed in our study. A limitation regarding the scope of our study was the fact that we primarily focused on how funding agencies require or recommend librarian involvement, rather than finding out how often librarians are actually involved in knowledge synthesis projects. It is possible that librarian participation occurs more frequently than the previously examined grants would suggest, meaning that their involvement is underrepresented in our study.

We also encountered several barriers while searching for knowledge synthesis grants. We searched primarily through institutional websites, some of which only show currently available grants on their public website and remove those that are closed, meaning we could not see past knowledge synthesis grants. This research may have benefited from a long-term analysis of the grant funding landscape in Canada, potentially over 12 or more months. Additionally, some institutions such as universities require an institutional login to view active and past grants, meaning we were unable to survey their grants for librarian involvement. Finally, we chose to focus on grants specifically intended for knowledge synthesis, rather than grants that could fund knowledge synthesis among other types of research. It is possible that we missed grants of this type that mention librarian involvement.

## Conclusion

Our study aimed to determine the degree to which the contributions of librarians in published knowledge synthesis are recognized and advocated for by research funding institutions in Canada. This was done by surveying knowledge synthesis project funding requirements from Canadian grant-funding agencies to see if librarians are mentioned as part of the funding requirements or as potential collaborators. We identified 14 grants that specifically fund knowledge synthesis research, 13 of which were provided by government agencies and one of which was provided by a Canadian non-profit agency. Of the 14 grants, only one, the “2022 Systematic and Scoping Review Grant Competition” from Alberta Health Services mentions librarian involvement as a requirement for funding [21], while none of the other grants mention librarians in any capacity within the eligibility criteria or in their descriptions of potential collaborators. In the granting organizations’ general guidelines on knowledge synthesis, only two out of seven organizations mentioned or recommended librarian involvement. This lack of acknowledgement of librarians’ contributions to knowledge synthesis research at the funding level comes despite the fact that CIHR, a leader in Canadian health sciences research, recommends that a librarian be part of the research team. However, in practice, neither the CIHR nor most other funding agencies actually require librarians to be involved in this research as a contingency for grant funding.

The health and medical grant funding landscape is complex, so there may be any number of reasons why librarian involvement is not required as a stipulation for knowledge synthesis research funding beyond what we have explored in this paper. Future research in this area could focus on the reasons why librarians are not brought into knowledge synthesis research projects and investigate the degree to which librarians are co-authors or acknowledged in published knowledge synthesis research.

## Data Availability

The list of organizations and grants identified throughout this study is available on the Open Science Framework (OSF) as CSV files using the link below: https://osf.io/4jbe8/?view_only=7f2b523aa2114e4f8da458fba67c61bf

## Footnotes

2 Scopus differs from typical database syntax in that quotation marks are interpreted as a ‘loose phrase’ as opposed to exact phrase searching. The typical function of quotation marks is represented in Scopus using curly brackets {} instead.

3 Type of knowledge synthesis was identified from the title and scope of the grant. Two grants included two types of knowledge synthesis, which are listed separately here. The total count of types of knowledge synthesis therefore does not match the count of grants identified in the study.

4 Government includes ministries, departments, research institutions, and agencies funded and organized by federal, provincial, or territorial branches of government.

5 SSHRC is the Social Sciences and Humanities Research Council; WSIB is the Workplace and Safety Insurance Board.

## Appendix - Grants included in study

**Table A1:** Grants Included in Study.

Institution	Institution Type	Jurisdiction	Grant Name	Librarian Involved*	Librarian Guidance**
Alberta Health Services	Government	Alberta	2022 Systematic and Scoping Review Grant Competition	Yes	Required [21]
Canadian Cancer Society	Non-Profit	Federal	CCS Accelerator Grant -Synthesis Grant	No	Not Mentioned [24]
Canadian Institutes of Health Research (CIHR)	Government	Federal	Indigenous COVID-19 Rapid Research Funding Opportunity	No	Strongly Recommended [22]
			Mental Health and Substance Use Responses to COVID-19	No	Strongly Recommended [22]
			Operating Grant: COVID-19 Knowledge Synthesis Network	No	Strongly Recommended [22]
			Operating Grant : SPOR –Guidelines and Systematic Reviews	No	Strongly Recommended [22]
Public Health Agency of Canada	Government	Federal	Public health measures for COVID-19 response research funding opportunity	No	Not Mentioned [25]
			Promoting Health Equity: Mental Health of Black Canadians Initiative –Knowledge Mobilization Network	No	Not Mentioned [26]
Social Sciences and Humanities	Government	Federal	Knowledge Synthesis Grant: Gender-Based	No	Not Mentioned [23]
Research Council of Canada (SSHRC)			Violence		
			Knowledge Synthesis Grants: Shifting Dynamics of Privilege and Marginalization	No	Not Mention[23]
Workplace Safety and Insurance Board (WSIB)	Government	Ontario	Occupational Asbestos Exposure and Development of Gastro-intestinal Cancers	No	Not Mention[28]
			Research and Grants Program	No	Not Mention[28]
			Systematic review: Occupational exposure to allergens and irritants and the development of occupational asthma	No	Not Mention[28]
WorkSafe BC	Government	British Columbia	Specific Priorities/Systematic Reviews Funding	No	Not Mention[30]

* “Librarian Involved” refers to whether a librarian or information professional is required as per the eligibility criteria of the grant.

** “Librarian Guidance” refers to whether the funding institution discusses librarians in any publication that relates to grant funding for a knowledge synthesis research project. This includes grant eligibility criteria, definitions of participants, guidance or best practices for knowledge synthesis research, and descriptions of grants, amongst other documents.
